# Repeated discomfort leading to a diagnosis of a voluminous right atrial myxoma in an adolescent patient: a case report

**DOI:** 10.11604/pamj.2022.41.25.32304

**Published:** 2022-01-11

**Authors:** Radohery Lovasoa Randriamanga, Etienne Rakotomijoro, Mefiarisoa Bodo Anna Rakotonirina, Muriel prudent

**Affiliations:** 1 Department of Pediatrics, University of Antananarivo, Antananarivo, Madagascar,; 2 Department of Polyvalent Medicine, University of Antananarivo, Antananarivo, Madagascar,; 3 Department of Cardiology, University of Antananarivo, Antananarivo, Madagascar,; 4 Department of Pediatrics, Hospital of Sens, Sens, France

**Keywords:** Adolescent, repeated discomfort, myxoma, right atrium, case report

## Abstract

Myxoma of the right atrium is rare, especially at young age. We report a voluminous right atrial myxoma in an adolescent admitted to the general pediatric ward for recurrent discomfort with deterioration of general condition, dyspnea and systemic inflammation. Transthoracic echocardiography revealed an intra-cardiac tumor 7.3 × 5.8 cm in diameter. After surgical excision, the post-operative outcome was favorable. Early diagnosis of cardiac myxoma is important to prevent the occurrence of complication. It should not be missed in a pediatric patient with repeated discomfort and systemic inflammation. Surgical excision remains the main treatment with favorable outcome.

## Introduction

Cardiac tumors in children are rare [1]. Cardiac myxomas are the most common primary benign cardiac tumors; they represent 50% of all primary cardiac tumors [2]. Their locations are often left intra-atrial. We report the case of a myxoma of the right atrium in a 17-year-old adolescent, revealed by a vague feeling of discomfort, deterioration of general condition and dyspnea.

## Patient and observation

**Patient information:** a 17-year-old male adolescent who, for 4 months, presented physical asthenia and weight loss of about 6kg and dyspnea on exertion, was hospitalized in the general pediatric ward for discomforts that occurred twice. No pathological history was found.

**Clinical findings:** on physical examination, a deterioration of the general condition was noted, the vital constants showed a tachycardia at 130 beats per minute. Blood pressure was 126/56mmHg. Cardiovascular examination revealed a 2/6 holo-systolic murmur of tricuspid valve regurgitation without irradiation, perceived peripheral pulses, hepato-jugular reflux and hepatomegaly. No rash at cutaneous examination.

**Figure 1:**
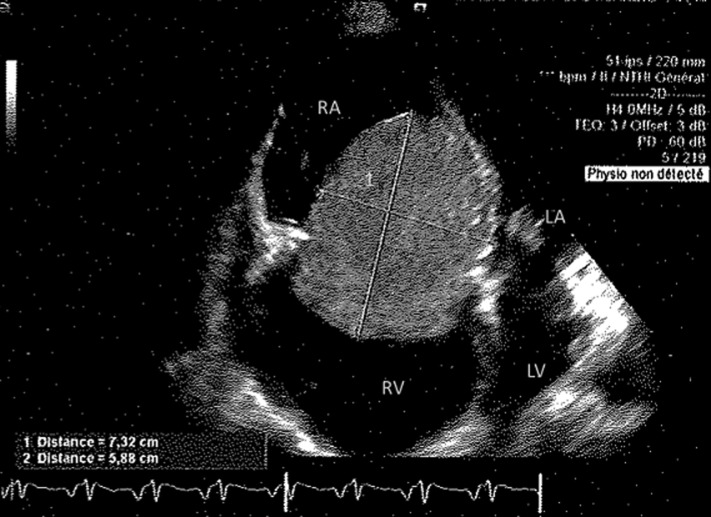
transthoracic echocardiography showing a right atrial mass passing through the tricuspid valve; 1: voluminous right atrial mass, RA: right atrium, RV: right ventricle, LA: left atrium, LV: left ventricle

**Figure 2:**
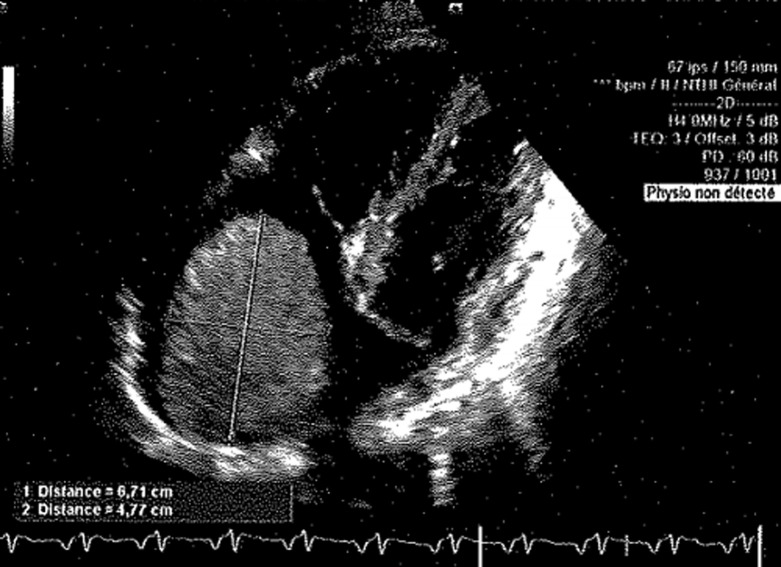
transthoracic echocardiography, other view showing the right atrial mass

**Diagnostic assessment:** the electrocardiogram (EKG) showed a regular sinus rhythm without any repolarization or conduction disturbances. Laboratory tests revealed anemia at 108g/l, a biological inflammatory syndrome with C-reactive protein (CRP) at 90 mg/l, sedimentation rate of red blood cells at 110mm and fibrinogen level at 6.34 g/l. The chest X-ray was strictly normal. A transthoracic echocardiogram showed a mass of 7.3 × 5.8cm in diameter hanging from the mobile right intra-atrial septum passing through the tricuspid valve with filling pressures increased to 10mmHg (Figure 1, Figure 2).

**Diagnosis:** the diagnosis of a tumor of the right atrium was made with a strong suspicion of myxomas.

**Therapeutic intervention:** the patient was referred to a cardiovascular and thoracic surgery department for emergency surgical excision. The first approach was a midline sternotomy. An extracorporeal circulation, between the ascending aorta and the two-vena cava, was established. A right atriotomy approach revealed a large right atrial tumor hanging from the fossa ovalis by an extremely sessile pedicle with a thrombus-like appearance; the entire tumor was resected. The postoperative course was favorable. The anatomo-pathological study of the piece confirmed the diagnosis of myxoma.

**Follow-up and outcomes:** follow-up echocardiography after 3 months did not show any recurrence of the myxoma. Clinically, the dyspnea had regressed with no signs of heart failure and no discomfort. The systemic inflammation had regressed as well. At 6 months follow-up, he remained well and there was no recurrence of the tumor.

**Patient perspective:** during his hospitalization and after the treatment, the patient and his family were delighted with the care he received and was optimistic about the outcome of his condition.

**Informed consent:** the patient and the family were informed about the case report, why the case was peculiar and the authors' interest in publishing his case. The family willingly gave informed consent to allow the authors to use his echo images for this case report.

**Patient's consent:** our patient and patient´s mother signed a written consent for publication of his clinical information and echo images.

## Discussion

Myxoma is the most common heart tumor, 90% of myxomas are diagnosed between the ages of 30 and 60. This tumor is rare in children [3] and common in women in their 30s [4]. However, our case shows that myxoma can be also found in adolescents and should be mentioned in the presence of a heart tumor during adolescence. As reported by Vinicius *et al*., presentation of cardiac myxoma can be systemic such as fever, weight loss, elevated inflammatory markers with high sedimentation rate and elevated CRP, anemia and leukocytosis [5] as we found in our patient. However, the clinical manifestations of myxoma of the right atrium are polymorphic and nonspecific: asymptomatic forms revealed by sudden death have been described with bulky forms that manifest at least one element of the clinical triad including impairment of general condition, embolic events, and syndromes of obstruction of the heart valve or cavity [6]. In our observation, with voluminous myxoma passing through the tricuspid valve, the clinical picture associated a deterioration of the general condition with repeated discomfort, signs of right heart failure, and a biological inflammatory syndrome, without embolic events. That emphasizes the importance of an early diagnosis by evoking cardiac myxomas, even at young patient, in front of trivial signs such as repeated discomfort with systemic inflammation. Indeed, it has been reported that delayed or undiagnosed cardiac myxoma can result in fatal complications due to cardiac obstruction or embolization of these friable tumors [7].

Thus, in front of suspected signs, the need for expedient diagnosis is primordial before complications occur. The radiologic and electrocardiographic signs of right atrium myxoma are nonspecific. Signs of congestion and pulmonary arterial hypertension, as well as tumor calcifications and distortion of the lower right border, have been described [8]; these abnormalities were not found in our patient. The localization of myxomas is eight times out of ten in the left atrium, in 10% in the right atrium, in 3% in the right ventricle and 3% in the left ventricle [1,2]. The localization of a myxoma in the right atrium, is quite rare especially at young age, as in our patient, and have been very rarely reported [9]. Our patient underwent a surgery under extracorporeal circulation. Indeed, this remains the treatment of choice for cardiac myxomas and studies have shown that operated patients benefit an equivalent survival rate to that of the general population [4]. Even if the 6 months follow-up of our patient have been good and with no recurrence of tumor, long-term ultrasound follow-up should be rigorous in order to detect any recurrence, especially in young patients [1].

## Conclusion

The peculiarity of the reported case of cardiac myxoma is the young age of onset and its location in the right heart passing through the tricuspid valve, which are very rarely reported. The symptoms can be trivial in adolescent but we emphasize the need of an early diagnosis before complications occur, by considering a cardiac myxoma in a patient with repeated discomfort and systemic inflammation and especially if associated with cardiac signs. The treatment of choice remains surgical excision and the prognosis is favorable.
